# Comparative analysis of Cd-responsive maize and rice transcriptomes highlights Cd co-modulated orthologs

**DOI:** 10.1186/s12864-018-5109-8

**Published:** 2018-09-26

**Authors:** Dan Cheng, Mingpu Tan, Haijuan Yu, Liang Li, Dandan Zhu, Yahua Chen, Mingyi Jiang

**Affiliations:** National Key Laboratory of Crop Genetics and Germplasm Enhancement, College of Life Sciences, Nanjing Agricultural University, Nanjing, China

## Abstract

**Background:**

Metal tolerance is often an integrative result of metal uptake and distribution, which are fine-tuned by a network of signaling cascades and metal transporters. Thus, with the goal of advancing the molecular understanding of such metal homeostatic mechanisms, comparative RNAseq-based transcriptome analysis was conducted to dissect differentially expressed genes (DEGs) in maize roots exposed to cadmium (Cd) stress.

**Results:**

To unveil conserved Cd-responsive genes in cereal plants, the obtained 5166 maize DEGs were compared with 2567 Cd-regulated orthologs in rice roots, and this comparison generated 880 universal Cd-responsive orthologs groups composed of 1074 maize DEGs and 981 rice counterparts. More importantly, most of the orthologous DEGs showed coordinated expression pattern between Cd-treated maize and rice, and these include one large orthologs group of pleiotropic drug resistance (PDR)-type ABC transporters, two clusters of amino acid transporters, and 3 blocks of multidrug and toxic compound extrusion (MATE) efflux family transporters, and 3 clusters of heavy metal-associated domain (HMAD) isoprenylated plant proteins (HIPPs), as well as all 4 groups of zinc/iron regulated transporter protein (ZIPs). Additionally, several blocks of tandem maize paralogs, such as germin-like proteins (GLPs), phenylalanine ammonia-lyases (PALs) and several enzymes involved in JA biosynthesis, displayed consistent co-expression pattern under Cd stress.

Out of the 1074 maize DEGs, approximately 30 maize Cd-responsive genes such as *ZmHIPP27*, stress-responsive NAC transcription factor (*ZmSNAC1*) and 9-cis-epoxycarotenoid dioxygenase (NCED, *vp14*) were also common stress-responsive genes reported to be uniformly regulated by multiple abiotic stresses. Moreover, the aforementioned three promising Cd-upregulated genes with rice counterparts were identified to be novel Cd-responsive genes in maize.

Meanwhile, one maize glutamate decarboxylase (ZmGAD1) with Cd co-modulated rice ortholog was selected for further analysis of Cd tolerance via heterologous expression, and the results suggest that ZmGAD1 can confer Cd tolerance in yeast and tobacco leaves.

**Conclusions:**

These novel findings revealed the conserved function of Cd-responsive orthologs and paralogs, which would be valuable for elucidating the genetic basis of the plant response to Cd stress and unraveling Cd tolerance genes.

**Electronic supplementary material:**

The online version of this article (10.1186/s12864-018-5109-8) contains supplementary material, which is available to authorized users.

## Background

Cadmium (Cd) is one of the most hazardous heavy metals, however it can be absorbed by the roots from the soil and transported to the aboveground parts, thus indirectly induces oxidative stress and negatively affects nutrient uptake and homeostasis, eventually causes stunted growth and reductions in productivity of crop plants [1]. Therefore, it is critically important to understand the mechanisms underlying Cd uptake, translocation and accumulation in plants.

Generally, Cd uptake and allocation is associated with a number of metal-regulated transporters including heavy metal ATPase (HMA), ATP-binding cassette transporters (ABC) subfamilies ABCC and ABCG, natural resistance-associated macrophage protein (Nramp), and zinc/iron regulated transporter protein (ZIP), which are indispensable for the homeostasis of essential metals [2–8].

Despite the identification of those genes, the underlying knowledge of molecular mechanisms for plant Cd tolerance is still fragmental. Using RNAseq profiling approach, thousands of genes involved in Cd uptake, translocation and detoxification have been identified in several plant species, such as radish [9], pakchoi [7], perennial herb [10], Zn/Cd hyperaccumulator *Sedum alfredii* Hance [11], fast growing Cd-resistant tree [12], dwarf Polish wheat [13], and sweet sorghum [14]. Recently, RNAseq studies allowed the identification of long non-coding RNAs and cis-natural antisense transcription in response to Cd stress in rice [15, 16].

Comparative intrageneric transcriptomic analyses have been used for revealing the mechanisms of Cd tolerance in plants [6, 7, 17]. For instance, RNAseq-based approach was utilized to unveil transcriptomic changes in maize seedlings roots under Cd stress [18, 19]. Of those DEGs in maize, some functional genes encoding stress and defense responses related proteins, transporters and transcription factors displayed great differential alteration in Mo17 and B73, and 115 genes were co-modulated in both genotypes across three time points [19].

Concerning the Cd responses in model cereals, the genome-wide RNAseq-based transcriptome profiling has been explored in rice [20–24]*.* As for the other model cereal maize, a few RNAseq-based transcriptomic studies focusing on Cd-responsive genes have been conducted [18, 19]. However, to the best of our knowledge, there is no report on the identification of universal cereal Cd-responsive genes, in other words, Cd co-modulated orthologs between maize and rice. Consequently, the common regulatory system for cereal crops in response to Cd is largely unknown and remains an essential issue to be addressed.

In the current study, we firstly implemented the Tophat-Cufflinks pipeline to identify early Cd-responsive DEGs in maize and rice seedlings roots. To ascertain our results of early transcriptomic response to Cd exposure, we compared Cd-regulated 5166 DEGs in maize with their 2567 counterparts in rice using plant orthologs annotation information, therefore the coordinated expression of cereal orthologs as well as maize paralogs was unveiled. After that, the Cd-responsive maize orthologs with synergistically Cd-regulated rice counterparts were queried against multiple stress common-responsive gene sets, and ~ 30 DEGs in the intersection are of particular interest, including those encoding HMAD isoprenylated protein ZmHIPP27, transcription factor ZmSNAC1, and key enzyme for ABA synthesis. Further Cd-tolerance assay in yeast and tobacco leaves indicated that ZmGAD1, which had Cd co-modulated rice ortholog, exhibited Cd tolerance in the host cells. These results could lead to a comprehensive understanding of the genetic basis of the plant response to Cd stress and open prospective for excavating novel genes and for the genetic improvement of plant tolerance to Cd stress.

## Methods

### Plant material and treatments

Seedlings of maize (*Zea mays* L. inbred line B73) were cultivated using a hydroponic system in a growth chamber under the conditions as described previously [24]. For Cd treatment, the trifoliate seedlings were transferred into fresh growing solutions containing 100 μM CdCl_2_. After 1 h of Cd treatment, maize seedlings roots were sampled for RNAseq analysis as described previously [20, 24].

### RNAseq libraries preparation and sequencing

Total RNA for RNAseq was extracted from maize seedling roots using a plant RNA kit (Omega, USA) according to the manufacturer’s instructions, and total RNA samples with two biological replicates were sent to Biomarker Corporation (www.biomarker.com.cn) for RNAseq library preparation and sequencing as described previously [24].

After the adaptor and low-quality sequences of pair-end reads were trimmed, a total of 38.71 Gb clean data from 6 cDNA libraries were obtained and all sequence reads have been deposited in the NCBI SRA datasets (www.ncbi.nlm.nih.gov/sra) under the accession number SRP115510. Over 80% of the clean reads had scores at the Q30 level (Additional file 1: Table S1).

### Mapping pair-end reads to the reference genome

The ‘Tuxedo’ packages TopHat-Cufflinks were utilized to process the RNAseq data [24, 25]. The B73 reference genome file ZmB73_RefGen_v2 and gene model annotation file ZmB73_5b_FGS.gff were downloaded from MaizeSequence (ftp://ftp.maizesequence.org/pub/maize/release-5b) directly. The expression values were represented by fragments per kilobase transcript per million reads mapped (FPKM), and the differential expression analysis of genes and transcripts across two conditions was performed using the Cuffdiff utility. Fold-change≥1.5 and q_value≤0.05 was set as the threshold to determine the DEGs between each set of compared samples as described previously [24].

MapMan (v3.6.0 RC1) was employed to annotate and subsequently visualize the stress-related DEGs on metabolic pathways [26].

For processing Cd-treated rice roots RNAseq data (www.ncbi.nlm.nih.gov/sra/SRP053169), the rice reference genome and gene model annotation files (MSU6) were downloaded from Illumina’s iGenomes project (support.illumina.com/sequencing/sequencing_software/igenome.html) directly, and the packages TopHat-Cufflinks were employed as described above.

### RNA isolation and cDNA synthesis for cloning gene coding sequence

Total RNA was extracted from plant material using the RNAiso Plus (TaKaRa Bio Inc., China) according to the manufacturer’s instructions. Approximately 2 μg of total RNA was reverse transcribed using oligo d(T)_16_ primer and M-MLV reverse transcriptase (TaKaRa). The synthesized cDNA was used for amplifying the coding sequences (CDS) of ZmGADs.

Transcript levels of randomly selected 10 genes were measured by qRT-PCR using a DNA Engine Opticon 2 real-time PCR detection system (Bio-Rad) with SYBR Premix Ex Taq (TaKaRa). The expression level of each target gene was normalized against that of *ZmActin* in maize. The primers used in the qRT-PCR experiments are listed in Additional file 2: Table S2.

### Yeast expression vector construction and cd-tolerance assay in yeast

To investigate whether ZmGADs confer Cd-tolerance in yeast, we subcloned the coding sequences of maize ZmGADs gene into the yeast expression vector *pYES2* with the primers listed in Additional file 2: Table S2.

Then the ZmGADs recombinant plasmid and *pYES2* empty vector (EV) were transformed into Cd-sensitive yeast *Δycf1* mutant cells using the lithium acetate transformation method, and the isogenic yeast wild-type BY4741(*MATa*; *ura3Δ0*; *leu2Δ0*; *his3Δ1*; *met15Δ0*) transformed with EV was used as a control [27–29].

Positive colonies were selected on synthetic dropout (SD) plates containing the appropriate selective markers (minimal medium lacking Uracil, designated as SD-Ura). Yeast strains expressing EV or ZmGADs were pre-cultured in SD-Ura liquid medium at 30 °C for 16 h. Pre-cultured cells were diluted to an OD_600_ of 1.0, and 10-μL aliquots were spotted onto SD-Ura agar medium with or without 40 μM CdCl_2_ in the presence of 2% galactose. The test plates were incubated at 30 °C for 3 days, then the growth of clones transformed with ZmGADs was compared with *Δycf1* cell*s* transformed with the EV on the same plates supplied with Cd ions [27, 29, 30].

Next, single clones that survived on the Cd-containing plates were selected and cultured in SD-Ura liquid medium supplemented with 30 μM CdCl_2_ and 2% galactose [27, 29, 30]. The growth of ZmGADs transformant was determined through measuring OD_600_ every 12 h, and the EV transformed Cd-sensitive *Δycf1* and the wild-type counterparts BY4741yeast cells were considered as controls in each experiment [31].

All drop-test experiments and growth turbidity assays were independently repeated at least three times.

### Agro-infiltration and cd response in tobacco leaves

The coding sequences of ZmGADs were amplified and inserted into the binary vector pCAMBIA1300-GFP. Agro-infiltration and Cd tolerance Assays was performed based on previous reports [32–35], with minor modifications.

*Agrobacterium tumefaciens* strain GV3101 was transformed with ZmGADs constructs and then grown in Luria-Bertani culture medium supplemented with appropriate antibiotics. After 36–48 h, *A. tumefaciens* cells were spun down by centrifugation, and re-suspended in Agro-infiltration buffer (10 mM MgCl_2_ and 10 mM 2-[N-morpholino] ethanesulfonic acid [MES], pH 5.6). The re-suspended *A. tumefaciens* cells were diluted and mixed with P19 silencing suppressor in a 1:1 to a final OD_600_ = 0.3 for each construct before infiltrating into the leaves of 3–4 week-old tobacco (*Nicotiana benthamiana*) plants.

After 3 d of agro-infiltration, tobacco leaves transiently expressing ZmGAD-GFP fusion proteins were analyzed using confocal fluorescence microscopy to monitor transformation. For fluorescence observations, patches were cut from tobacco leaves 3 d after agro-infiltration and used for confocal imaging on a Zeiss LSM 710 confocal laser scanning microscope. RFP-H2A, localized in the nucleus, was used to mark the nuclei [36]. GFP fluorescence was excited by the 488 nm line of an argon laser, and emissions were detected between 500 and 530 nm.

After 3 days of agro-infiltration, the needle hole in the leaves expressing ZmGAD- GFPs were re-infiltrated with 500 μM Cd(NO_3_)_2._ The infected leaves were photographed at 4 days post-treatment.

Each experiment was repeated at least three times with a minimum of 10 infected leaves. Leaf regions transiently expressing EV were used as a control.

## Results

### Functional characterization of early cd-responsive differentially expressed genes (DEGs) in maize roots

To investigate transcripts that were specifically regulated in short-term Cd stressed maize roots, RNAseq data from replicated samples were processed through TopHat-Cufflinks pipeline to perform pair-wise comparisons between 1 h Cd-treated (Cd1h) and untreated (ck1h) maize seedlings (Additional file 1: Table S1).

Using a moderate cutoff (fold change> 1.5 and q_value≤0.05), a total of 5166 genes were identified as being early Cd-responsive differentially expressed genes (DEGs), of which 3715 were Cd-induced and 1451 were Cd-repressed in maize seedlings roots (Additional file 3: Table S3)**.** However, only 239 DEGs were in the intersection of these 5166 and the available 768 Cd-responsive genes in B73 or Mo17 across three time points reported previously [19] (Additional file 3: Table S3). The expression pattern of randomly selected 10 DEGs was basically consistent with that of qRT-PCR validation (Additional file 4: Table S4), which suggested that DEGs resulted from RNAseq are credible for further analysis.

To gain insights into the functionality of the 5166 DEGs that are likely to be associated with the Cd response, all of these Cd-responsive transcripts were functionally grouped and visualized in the candidate pathway networks with MapMan software.

**Fig. 1:**
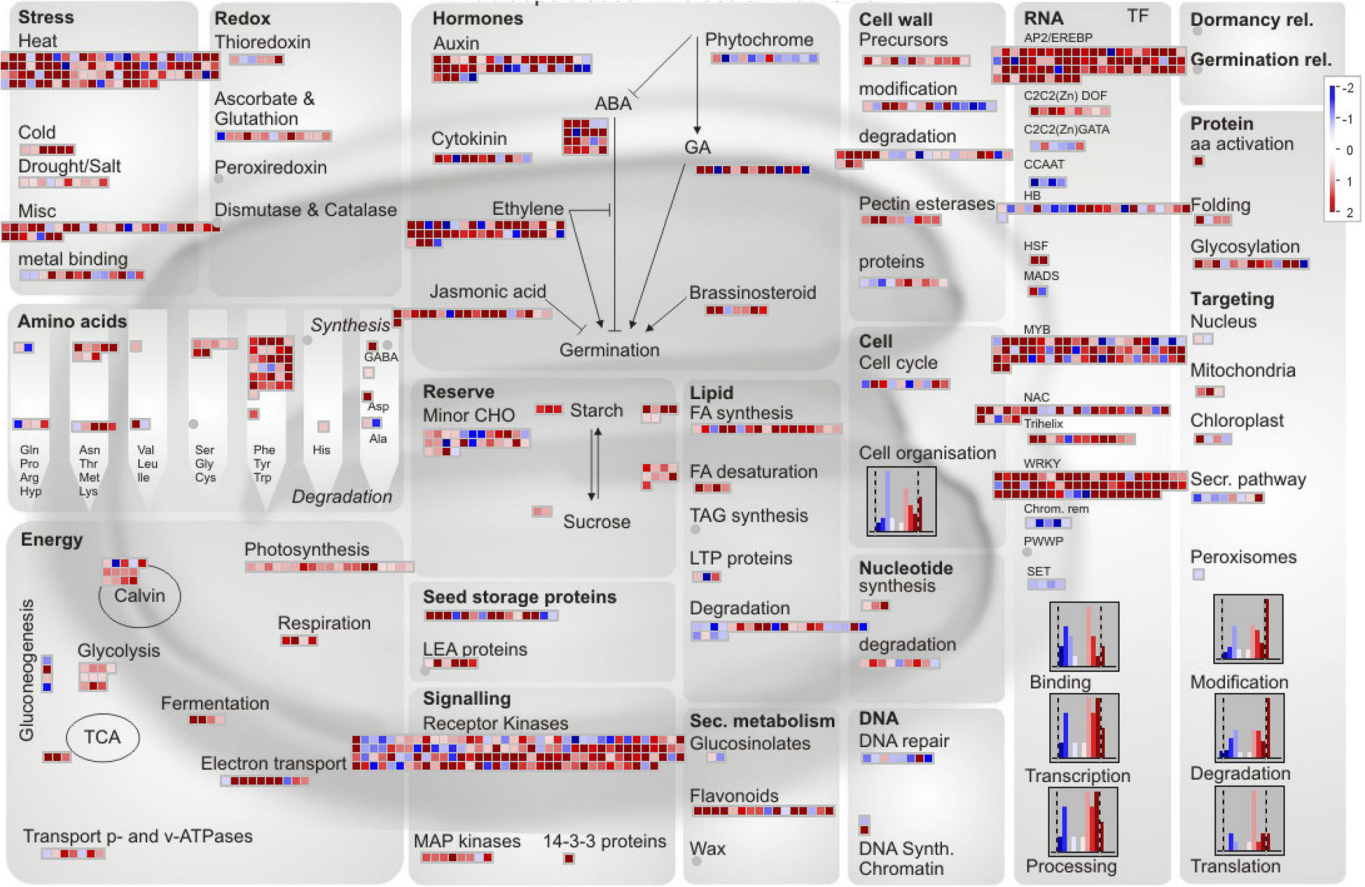
Global view of 5166 DEGs involved in diverse metabolic pathways in maize roots under Cd stress. Among the 4689 mapped data points of 5166 DEGs, 1767 data points were visible on the metabolic pathways (Arabidopsis seed-Molecular Networks) using MapMan software. The colored boxes indicate the Log2 ratio of Cd1h/ck1h

Among the DEGs within the ‘TF’ group, 9 members of C2C2(Zn) DOF zinc finger family, 11 HSFs (including those in the subcategory “heat” of ‘Stress’ group), 11 members of Triple-Helix TFs family, and the majority of EREBP and NAC as well as WRKY family TFs were upregulated in response to Cd (Fig. 1, Table 1).

**Table 1:** The function classification of maize DEGs with rice orthologs

Categories	Gene IDs					cluster	Cd-regulation
Abiotic stress							
HIPP, heavy metal-associated domain (HMAD) isoprenylated plant protein							
	ZM2G085086	ZM2G008290	ZM2G086163				UP
HSP, heat shock protein	**ZM2G056039**	**ZM2G310431**	**ZM2G340251**	**ZM2G428391**	**ZM5G802801**	MCL144	UP
	**ZM2G046382**	**ZM2G158232**	**ZM2G306679**			MCL173	UP
	**ZM2G012631**	**ZM2G069651**	**ZM2G112165**			MCL313	UP
	ZM2G070863	ZM2G083810	ZM2G098167	ZM2G117836			UP
	ZM2G149647	ZM2G335242	ZM2G360681	ZM2G375517	ZM2G007729		UP
HSF, heat shock transcription factor	**ZM2G098696**	**ZM2G164909**				MCL2494	UP
	ZM2G059851	ZM2G118453	ZM2G301485				UP
DNAJ protein	**ZM2G010000**	**ZM2G031637**	ZM2G010871		ZM2G029385	MCL1373	UP
	**ZM2G039886**	**ZM2G119316**	ZM2G023786			MCL1040	UP
	**ZM2G028218**	**ZM2G134917**	ZM2G086841			MCL685	UP
cold-responsive CaLB domain	ZM2G032766						UP
drought/dehydration-responsive	**ZM2G077036**	**ZM2G128179**	ZM2G014066	ZM2G069018	ZM2G181551	MCL561	UP
GLP, germin-like protein	***ZM2G093622***	***ZM2G093606***	***ZM2G093554***	***ZM2G157364***		MCL23	UP
	**ZM2G093076**	**ZM2G149714**	**ZM2G049930**	**ZM2G071390**	ZM2G178817	MCL23	UP
	***ZM2G072965***	***ZM2G176798***	***ZM2G170857***	ZM2G045809	ZM2G115491	MCL23	UP
cyclase/dehydrase family	ZM2G144224	ZM2G047677					down
MAP kinases signaling	ZM2G053987	ZM2G344388					UP
ABA synthesis and signaling							
AAO, abscisic aldehyde oxidase	ZM5g899851						UP
NCED, 9-cis-epoxycarotenoid dioxygenase		**ZM2g408158**	**ZM2g417954**	ZM2g014392		MCL13945	UP
ABA signaling	ZM2g479760	ZM2g114153	ZM2g046782	ZM2g106622			UP
Jasmonate synthesis							
LOX, lipoxygenase	***ZM2G109056***	***ZM2G109130***	**ZM2G102760**			MCL163	UP
AOS, allene oxidase synthase	***ZM2G072653***	***ZM2G376661***	**ZM2G033098**			MCL3533	UP
	**ZM2G002178**	**ZM2G067225**				MCL3596	UP
AOC, allene oxidase cyclase	ZM2G077316						UP
OPR, 12-oxophytodienoate reductase	**ZM2G000236**	**ZM2G087192**				MCL166	UP
Ethylene synthesis and signaling							
ethylene synthesis	**ZM5g894619**	**ZM2g164405**				MCL4831	UP
ethylene signaling	**ZM2g438202**	**ZM2g068967**	ZM2g020016	ZM2g051135	ZM2G061487	MCL12147	UP
	ZM2g123119	ZM2G174347	ZM2g381441	ZM2g474326	AC233933.1_FG001		UP
ethylene receptor	AC194965.4_FG001						down
Cytokinin synthesis and degradation							
cytokinin synthesis	ZM2g104559						down
cytokinin degradation	ZM2g024476	ZM2g348452					UP
Cell wall							
CW precursor synthesis	**ZM2G110558**	**ZM2G042179**	ZM5G862540			MCL7320	UP
CW modification	ZM2G070271	ZM2G114322			ZM2G332412		down
CW extensin	**ZM2G099491**	**ZM2G070322**				MCL13774	UP
Redox							
respiratory burst	**ZM2G065144**	**ZM2G441541**				MCL365	UP
AsA and GSH cycling	ZM2G134708	ZM2G141376					UP
glutaredoxins	**ZM2G178886**	**ZM2G311898**	ZM2G148387			MCL631	UP
GST, glutathione S-transferases	ZM2G052625	ZM2G308687	ZM2G161827	ZM2G161891	ZM2G044383		UP
	ZM2G025190	ZM2G146913	ZM2G175134	ZM2G475059			UP
minor CHO metabolism							
raffinose family synthases	**ZM2G077181**	**ZM2G340656**	ZM2G165919			MCL3328	UP
trehalose synthesis	**ZM2G014729**	**ZM2G117564**	ZM2G112830			MCL11250	UP
Development							
patatin-like storage proteins	**ZM2G114036**	**ZM2G414047**			ZM2G117378	MCL12549	UP
	**ZM2G091956**	**ZM2G124921**				MCL12894	UP
LEA, late embryogenesis abundant	ZM2G099003	ZM2G093418					UP
Secondary metabolism							
isoprenoids	ZM2G150367						UP
phenylpropanoids	**ZM2G060210**	**ZM2G108714**				MCL12214	UP
	**ZM2G114918**	**ZM2G061806**	ZM2G064969	ZM2G140996		MCL16463	UP
	ZM2G165192	ZM2G125448	ZM2G362298	ZM5G882427			UP
lignin biosynthesis. PAL phenylalanine ammonia-lyase		***ZM2G029048***	***ZM2G334660***	**ZM2G170692**		MCL71	UP
		**ZM2G081582**	**ZM2G063917**	**ZM2G118345**		MCL71	UP
lignin biosynthesis. Others	ZM2G167613	ZM2G100158	ZM2G125448	AC234163.1_FG002			UP
flavonoids and anthocyanins	ZM2G117246	ZM2G382785	ZM2G099467	ZM5G881887	ZM2G051683		UP

DEGs within the same orthologs group are in bold, while paralogous DEGs are in italic. DEGs underlined are also Cd-responsive reported previously [19]. Plant orthologous group prefix APK_ORTHOMCL is abbreviated as MCL

Of the transcripts mapped to ‘Hormones’ category, 17 genes (including 7 lipoxygenase LOX, 5 allene oxidase synthase AOS, one allene oxidase cyclase and four 12-oxophytodienoate reductases OPR) for jasmonate synthesis, and 6 genes for brassinosteroid metabolism and signaling were also upregulated by Cd treatment. With regard to ‘Stress’ response, all 6 genes in response to cold and almost all genes responsive to drought/salt were upregulated post Cd treatment. In addition, almost all pectin esterases and genes for cell wall precursor synthesis in ‘Cell wall’ category were upregulated in response to Cd (Fig. 1, Table 1).

In contrast, all mapped 4 nuclear transcription factors (NF-Y) of CCAAT box binding factor family, 5 chromatin remodeling factors, and 4 members in SET-domain transcriptional regulator family were uniformly downregulated by Cd treatment (Fig. 1, Additional file 3: Table S3).

### Conserved cd-responsive orthologous genes in maize and rice roots

To date, no comprehensive list of maize genes orthologous to rice genes involved in Cd stress response is available. Therefore, the global comparison of the DEGs identified in the short-term Cd treated maize and rice roots (NCBI-SRA SRP053169, Additional file 5: Table S5) was performed with the aid of plant model organism orthologs annotation [37, 38].

This comparison output 1074 Cd-responsive maize orthologs of 981 rice genes, which can be categorized into 880 plant orthologous groups (APK_ORTHOMCL abbreviated as MCL, Table 1, Additional file 6: Table S6). For the 1074 maize DEGs having differentially expressed counterparts in rice, 939 were upregulated and 135 downregulated by Cd stress in maize roots (Table 2, Additional file 6: Table S6, Additional file 7: Figure S1). Moreover, 80 of them are in the list of 768 Cd-responsive genes in B73 or Mo17 roots under various Cd pressures [19]. Conversely, 994 maize DEGs with rice counterparts were not stated as Cd-responsive genes previously (Additional file 6: Table S6). Among the 80 universal Cd-responsive DEGs, 4 GLP members in group MCL23, 2 patatin-like members of MCL12549, and two 12-oxo-phytodienoic acid reductases (ZM2G000236 and ZM2G087192 designated as ZmOPR2 and ZmOPR5, respectively) in cluster MCL166 were also significantly upregulated in both maize genotypes (Table 1) [19].

**Table 2:** Cd-regulated DEGs of orthologous transporters in maize and rice roots

BinName		Maize			Rice		Plant Orthologous Groups
	Gene ID	Log2FC	Annotation	Gene ID	log2FC	Annotation	APK_ORTHOMCL
Metal binding, chelation and storage							
	ZM2G085086	2.43	ZmHIPP27, HMAD isoprenylated plant protein	Os04g17100	3.62	heavy metal-associated domain (HMAD), OsHIPP42	MCL4978
	ZM2G008290	3.75	ZmHIPP35	Os10g30450	0.93	OsHIPP35	MCL1350
	ZM2G086163	4.51	ZmHIPP36	Os03g05750	7.38	OsHIPP36	MCL16487
ABC transporters and multidrug resistance systems							
	ZM5G874955	5.87	ABC transporter G family member 40	Os01g42380	2.15	ABCG36/OsPDR9, pleiotropic drug resistance protein	MCL2
	ZM5G892675	5.00	ABC transporter G family member 36	Os01g42410	2.59	ABCG37/OsPDR8	MCL2
	ZM2G003411	2.64	ABC transporter G family member 39	Os02g11760	3.10	ABCG39/OsPDR7	MCL2
	ZM2G366146	2.32	ABC transporter G family member 42	Os01g42370	1.68	ABCG35/OsPDR11	MCL2
	ZM2G143139	2.22	ABC transporter G family member 37	Os08g29570	4.72	ABCG44/OsPDR17	MCL2
	ZM2G415529	1.27	ABC transporter G family member 43-like				MCL2
	ZM2G391815	1.23	ABC transporter G family member 34				MCL2
Divalent cations transporters							
	ZM2G118821	−0.92	ZmIRT1, zinc transporter 10	Os03g46470	−2.58	OsIRT1, Iron-regulated transporter	MCL3982
	ZM2G045849	−1.05	ZmZIP3, zinc transporter 1	Os05g39540	−1.93	OsZIP9, ZRT/IRT-like protein	MCL386
	ZM2G015955	−0.94	ZmZIP7, zinc transporter 4	Os06g37010	−1.39	OsZIP10	MCL2136
	ZM2G047762	−0.81	ZmZIP9, zinc transporter 5	Os05g39560	−1.98	OsZIP5	MCL14115
	ZM2G144083	−1.83	ATP dependent copper transporter	Os04g46940	−0.99	OsHMA5, heavy metal P-type ATPase	MCL2236
Amino acid transporters							
	ZM2G164814	2.35	amino acid carrier	Os01g66010	1.48	amino acid transporter	MCL399
				Os05g34980	0.99	amino acid transporter	MCL399
	ZM2G157168	3.72	amino acid permease 2	Os12g08090	2.73	amino acid transporter	MCL2026
	ZM2G433162	1.46	amino acid permease 2	Os12g08130	1.65	amino acid transporter	MCL2026
MATE efflux family and other transporters							
	ZM2G031938	1.37	protein DETOXIFICATION 40	Os03g37490	0.99	PEZ1, Phenolic Efflux Transporter	MCL409
	ZM2G170128	0.92	transparent testa 12 protein				MCL409
	ZM2G151903	1.93	Protein DETOXIFICATION 21	Os12g03260	1.53	MATE efflux family protein	MCL636
	ZM2G079127	2.00	Protein DETOXIFICATION 21				MCL636
	ZM2G006212	3.31	protein DETOXIFICATION 49	Os02g45380	2.76	MATE efflux family protein	MCL1103
	ZM2G080992	2.96	protein DETOXIFICATION 49	Os04g48290	1.14	MATE efflux family protein	MCL1103
	ZM2G135175	2.86	protein DETOXIFICATION 49				MCL1103
	ZM2G119970	5.08	adenine/guanine permease AZG1	Os05g26840	1.05	permease domain	MCL6695
	ZM2G358791	2.03	adenine/guanine permease AZG2	Os11g24060	1.01	permease domain	MCL7794
	ZM2G068220	0.80	adenine/guanine permease AZG2				MCL7794

Plant orthologous group prefix APK_ORTHOMCL is abbreviated as MCL. Those genes underlined are also Cd-responsive DEGs in previous report [19]

To further explore the common regulatory mechanisms in maize and rice under Cd stress, these Cd-responsive orthologs were investigated their involvement in various metabolic pathways. According to the global function view of these Cd-responsive orthologs in MapMan, the majority of them were upregulated in response to Cd, whether in maize or in rice roots (Additional file 7: Figure S1). Apparently, the uniformly upregulated DEGs were enriched in abiotic stress response (heat, cold and drought/salt), hormone metabolism and signaling (ABA, ethylene and JA), cell wall precursors biosynthesis, as well as several TFs families (e.g. EREBP, NAC and WRKY).

Interestingly, the majority of mapped maize Cd-responsive orthologs can be categorized into orthologous clusters and exhibit group co-regulated manner. Particularly, Cd-induced germin-like protein (GLP) genes are concentrated on MCL23, while all 6 Cd-induced members of phenylalanine ammonia-lyase (PAL) belong to group MCL71 (Table 1, Fig. 1). Moreover, 9 GLPs of MCL23 cluster on chromosome 4. Out of this subset of 9 GLPs, both 4 members (ZM2G093622, ZM2G093606, ZM2G093554 and ZM2G157364) and other 3 members (ZM2G072965, ZM2G176798 and ZM2G170857) are sorted in tandem, and one gap (about 127 kb) separated these two GLPs blocks. Similarly, two PALs (ZM2G029048 and ZM2G334660) and two patatin-like storage proteins (ZM2G124921 and ZM2G091956) are tandem paralogs on chromosome 5, chromosome 2, respectively, while two LOXs (ZM2G109056 and ZM2G109130) together with two AOSs (ZM2G072653 and ZM2G376661) are tandem paralogs on chromosome 1. Additionally, another two Cd-responsive patatin-like storage proteins (ZM2G114036 and ZM2G414047) reported previously [19] are reverse tandem paralogs on chromosome 1 (Table 1).

According to the transport overview, the orthologs of mapped transporters displayed concordant expression pattern in Cd-treated maize and rice roots (Table 2). One orthologous group of PDR-type ABC transporters (MCL2), two groups of amino acid transporters, and 3 groups of MATE efflux family transporters as well as 3 groups of HMAD isoprenylated proteins (HIPPs) were all rapidly upregulated in Cd-treated maize and rice roots. However, all 4 orthologous groups of ZRT/IRT-like transporter proteins (ZIPs) and one orthologous group of Cu transporter were downregulated concomitantly by Cd stress in both species, indicating a certain level of conservation in Cd response (Table 2, Additional file 3: Table S3, Additional file 5: Table S5).

However, it is noteworthy that 25 of the 28 Cd-responsive maize transporters with co-modulated rice orthologs were not demonstrated as Cd-responsive genes previously (Table 2).

### Common stress-responsive genes in maize

To ascertain whether these conserved Cd-responsive maize genes are involved in diverse stress response, they are compared with those identified in previous RNAseq analysis of maize seedlings subjected to drought, salinity and cold [39, 40]. Remarkably, out of 1074 Cd-responsive maize DEGs with rice counterparts, about 30 genes are also in the list of DEGs response to these abiotic stresses, and they can be grouped into 5 categories. With respect to those located in ‘abiotic stress’, ZmHIPP27 and Lea5-D-like are particularly noteworthy. Regarding ‘transcription factors’, ZmSNAC1, 6 ERFs including two DREBs, two zinc finger proteins, and WRKY40 were all common stress-responsive genes. Among those related to ‘Phytohormone and signaling’, *vp14* encoding NCED is responsible for ABA biosynthesis, meanwhile, auxin-induced in root cultures AIR12, ZIM transcription factor, and gibberellin receptor GID1 are involved in auxin, JA, and GA signaling, respectively. Moreover, *vp14* has been repetitiously identified to be common stress-responsive maize gene in previous reports (Table 3).

**Table 3:** The expression of common stress-responsive maize genes and their rice orthologs in plant roots exposed to Cd treatment

Categories	Maize			Rice		Plant Orthologous Groups
Gene ID	Log2FC	maize Annotation	Gene ID	log2FC	rice Annotation	ORTHOMCL
Abiotic stress						
ZM2G085086	2.4	ZmHIPP27, HMAD isoprenylated protein	Os04g17100	3.6	heavy metal-associated domain (HMAD) OsHIPP42	MCL4978
ZM2G099003	2.6	Lea5-D-like	Os01g21250	2.3	LEA, late embryogenesis abundant protein	MCL16656
ZM2G012631	0.9	HSP90–2	Os08g39140	1.5	heat shock protein	MCL313
ZM2G032766	2.7	CaLB domain protein	Os08g44850	1.2	C2 domain containing protein	MCL5451
Transcription factors						
ZM2G347043	3.4	NAC49; ZmSNAC1	Os03g60080	3.4	SNAC1, stress-responsive NAC 1	MCL15794
ZM2G069146	4.2	dehydration-responsive element-binding protein	Os09g35030	3.5	OsDREB1A	MCL12934
ZM2G061487	2.3	DRE binding factor 1	Os08g31580	1.4	ERF, ethylene-responsive transcription factor	MCL17488
ZM2G174347	1.4	ERF	Os05g41780	1.0	AP2 domain containing protein	MCL5811
ZM2G068967	1.9	ERF	Os04g52090	1.4	OsAP2–39	MCL12147
ZM2G438202	2.0	ERF				MCL12147
ZM2G474326	2.0	ERF	Os01g54890	2.7	OsERF922	MCL13082
ZM2G093270	1.4	PLATZ transcription factor	Os10g42410	3.7	zinc-binding protein	MCL17475
ZM2G101058	1.3	GATA28	Os10g40810	0.7	GATA zinc finger domain protein	MCL1807
ZM2G361210	6.7	C2H2 Zinc finger protein ZAT11	Os03g60570	5.7	ZFP15, C2H2 zinc finger protein	MCL13769
ZM2G061626	5.3	C2H2 zinc finger protein	Os03g60560	7.5	ZFP182, C2H2 zinc finger protein	MCL13770
ZM2G158328	3.3	WRKY40	Os01g60600	2.0	WRKY108	MCL17062
Phytohormone signaling						
** ZM2G014392**	4.6	vp14, 9-cis-epoxycarotenoid dioxygenase	Os03g44380	3.4	OsNCED3, 9-cis-epoxycarotenoid dioxygenase	MCL9668
ZM2G427451	0.8	AIR12	Os08g41290	0.7	AIR12, auxin-induced in root cultures	MCL9576
ZM2G036351	5.3	ZIM transcription factor	Os03g08330	3.5	OsJAZ10, ZIM domain containing protein	MCL14008
ZM2G173630	1.6	GID1 (GA-insensitive dwarf)	Os05g33730	−0.7	gibberellin receptor GID1L2	MCL4519
ZM2G033846	3.3	caltractin	Os03g19720	3.7	EF hand family protein	MCL5182
ZM2G312661	1.8	Calmodulin	Os03g21380	1.5	OsCML27, Calmodulin-related protein	MCL16352
Cell wall						
ZM2G110558	1.1	UDP-glucuronate 4-epimerase	Os02g54890	0.9	UDP-glucuronate 4-epimerase	MCL7320
ZM2G042179	0.8	UDP-glucuronate 4-epimerase				MCL7320
ZM2G015886	0.7	Cellulose synthase D4	Os12g36890	0.9	CSLD4, cellulose synthase-like family D,	MCL85
ZM2G135743	1.0	glycogenin-like starch initiation protein	Os01g65780	−0.9	glycosyl transferase	MCL1732
Posttranslational modification						
ZM2G172230	2.4	CaseinoLytic Protease ClpD, chloroplastic	Os02g32520	1.3	OsClpD1, early responsive to dehydration ERD1	MCL2599
ZM2G328785	3.5	ZmPK1, receptor protein kinase	Os11g03820	−1.3	S-locus-like receptor protein kinase	MCL8097
ZM2G359986	1.8	Wall-associated receptor kinase-like 20	Os05g25390	2.4	tyrosine protein kinase	MCL7431
ZM2G443509	−0.7	protein phosphatase 2C	Os04g33080	−1.2	protein phosphatase 2C	MCL2430

The DEGs identified in both previous reports [39, 40] are in bold, and the DEGs underlined are also Cd-responsive in previous report [19]

Taking ‘Cell wall’ into account, two common stress-responsive genes encode UDP-glucuronate 4-epimerase and one gene code cellulose synthase. Besides these 4 categories, the category ‘Posttranslational modification’ containing 4 common stress-responsive genes is of particular interest.

Among the 30 common stress-responsive genes, only ZM2G172230 encoding CaseinoLytic Protease (ClpD) and ZM2G328785 coding receptor protein kinase (ZmPK1), both in the category ‘Posttranslational modification’, were also reported to be steadily induced by Cd treatments across several time points (Table 3) [19].

### ZmGADs confer cd tolerance in cd-sensitive yeast mutant

To unveil novel Cd-tolerant genes from the Cd-responsive orthologs in maize, the key enzyme responsible for γ-aminobutyric acid (GABA) synthesis named glutamate decarboxylase (GAD) was selected, since Cd-regulated GAD orthologs in maize and rice were mapped to GABA biosynthesis pathway coincidentally (Fig. 1, Additional file 7: Figure S1).

It is noteworthy that ZmGAD1 (ZM2G098875) and its ortholog OsGAD3 (Os03g13300), within the orthologs group MCL1496, were all upregulated by Cd stress, with the Log_2_FC of 2.7 and 0.84, respectively (Additional file 3: Table S3, Additional file 5: Table S5, Additional file 6: Table S6). These results illustrate that key genes involved in GABA biosynthesis are uniformly stimulated in maize and rice roots under Cd stress.

To establish a close link between GABA and Cd-tolerance, maize ZmGAD1 and ZmGAD2 (ZM5G826838) within the same orthologous group MCL1496 were cloned into the yeast expression vector pYES2 and retransformed to Cd-sensitive yeast *Δycf1* cells to test whether ZmGADs can complement *Δycf1* phenotype, thus confirming their functionality of Cd-tolerance.

**Fig. 2:**
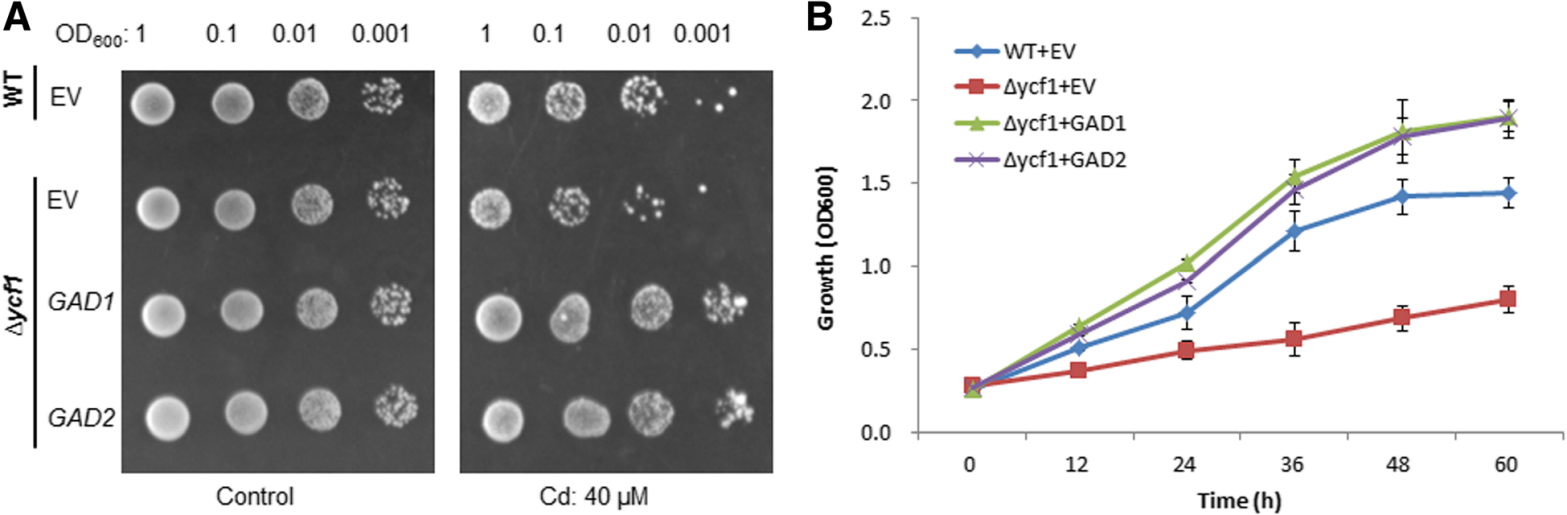
Maize *ZmGAD1*and *ZmGAD2* conferred Cd tolerance in yeast. **a** The survival test of yeast strains transformed with *ZmGADs* on SG-Ura agar medium supplemented with 40 μM CdCl_2_ in the presence of 2% galactose. The photographs were taken 3 days post incubation, with three independent experiments. **b** Time-dependent growth curves of yeast transformed with *ZmGADs* in SG-Ura liquid medium with 30 μM CdCl_2_ and 2% galactose. The growth of the yeast cells was monitored at OD_600._ Data are presented as means ± SE (*n* = 3). Error bars indicate SE of three independent biological experiments. Statistical analysis was performed using SPSS20.0 software

The dilution spot tests and turbidity growth assays showed that the *Δycf1* mutant transformed with *ZmGADs* exhibited dramatically enhanced growth when compared with Δ*ycf1* cells transformed with the pYES2 empty vector (EV) under the Cd pressure (Fig. 2a, b).

### ZmGADs confer cd tolerance in tobacco cells via a transient assay

**Fig. 3:**
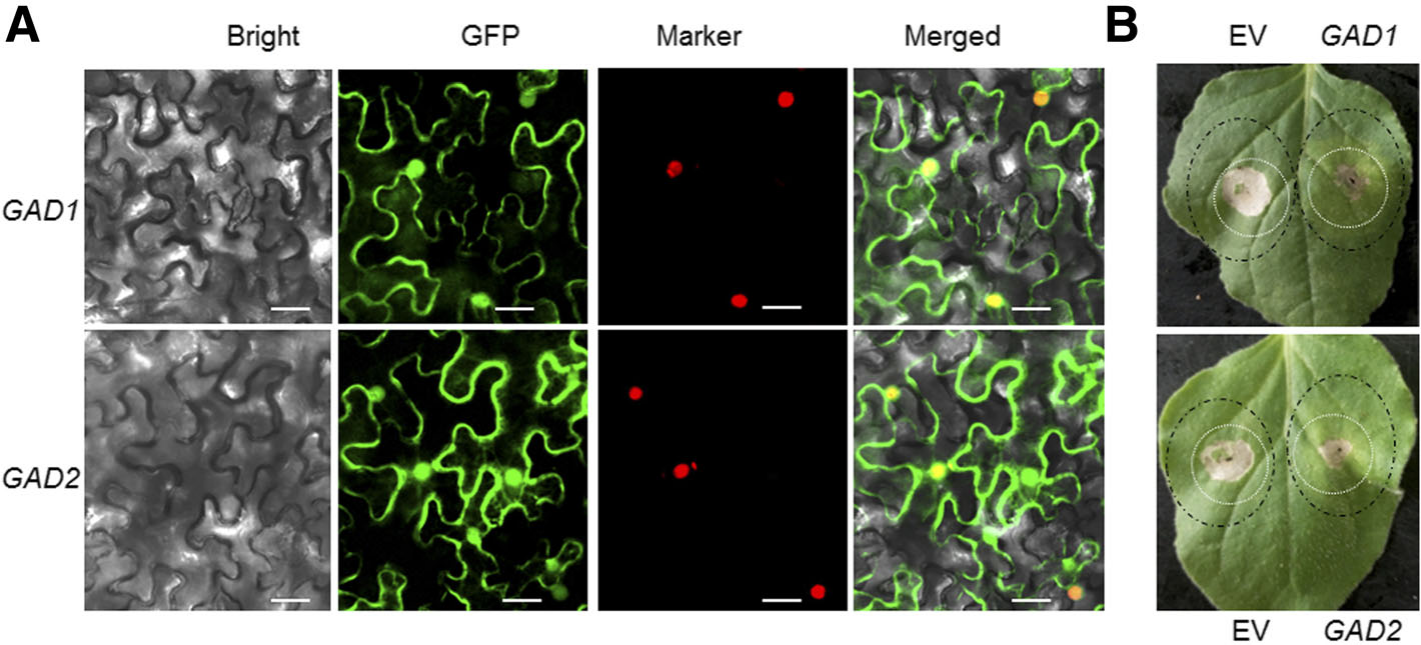
Transient expression of maize *ZmGAD1* and *ZmGAD2* in tobacco leaves led to increased tolerance to Cd*.* **a** Nuclear localization of GFP-tagged ZmGAD1 and ZmGAD2 fusion proteins in *N. benthamiana*. Epidermal cells of *N. benthamiana* leaves transiently expressing GFP fusion proteins were observed using confocal microscopy at 4 days post agro-infiltration. RFP-H2A, localized in the nucleus, was used to indicate the nuclei [36]. Scale bars = 50 μm. **b** Lesions were photographed at 4 days post Cd treatment on EV transformed control regions (left half-leaf) and regions transient expressing ZmGADs (the right part). Cd-infiltrated areas are indicated by the internal white circles, whereas agro-infiltrated areas are indicated by the larger black circles

To assess the validity of the results from yeast complementation assay, tobacco leaves-based *in planta* transient analysis was used. To ascertain whether these Cd tolerant genes confer Cd tolerance *in planta*, we initially introduced two GAD-green fluorescent protein (GFP) fusion pSuper-1300 constructs into tobacco leaf cells by agro-infiltration. The results showed that GFP-tagged ZmGAD1 and ZmGAD2 fused proteins accumulated in tobacco leaves 3 d post infiltration, meanwhile, the subcellular localization assay indicated that all these 2 GFP fused proteins were localized in cytoplasm and nucleus (Fig. 3a).

To assess the functional relevance of GADs in maize against Cd stress, tobacco leaves transiently expressing them validated above were treated by infiltrating Cd solution, and the leaf regions transiently expressing EV were used as a control. The results showed that the EV transformed control regions showed chlorosis and lesions 4 d after Cd treatment, indicative of a symptom of Cd toxicity. However, lesions on leaves transiently expressing ZmGADs were significantly reduced than those on leaves expressing EV after Cd treatment **(**Fig. 3b).

These results, confirmed in yeast and tobacco leaves through ectopic expression, suggest that maize GADs confer Cd tolerance.

## Discussion

Coordinated expression of transporters orthologs indicate their conserved function in Cd-stressed maize and rice.

To further explore the common regulatory mechanisms in maize and rice under Cd stress, we compared Cd-regulated 5166 maize DEGs with their 2567 counterparts in rice (Additional file 3: Table S3, Additional file 5: Table S5). As anticipated, the majority of the orthologous DEGs in maize and rice showed coordinated expression pattern after Cd treatment. Notably, 994 out of 1074 maize DEGs with rice counterparts were not stated as being Cd-responsive genes previously (Table 2, Additional file 6: Table S6).

Among the co-modulated transporters in Cd-treated maize and rice roots, it is noticeable that Cd-responsive HIPPs were categorized into 3 groups of co-upregulated orthologs (Table 2). HIPP metallochaperones, containing a metal binding domain, play a pivotal role in heavy metal homeostasis and detoxification, especially in Cd tolerance [41–43]. Overexpression of Arabidopsis *AtHIPP06* [44] or *AtHIPP26* [45] conferred Cd tolerance to transgenic plants, however, the triple knockout mutant *hipp20/21/22* was more sensitive to Cd [43]. Interestingly, *AtHIPP06* and *AtHIPP26* were distributed to two Cd co-modulated orthologous groups containing *ZmHIPP35* and *ZmHIPP27*, respectively.

The other notable orthologous transporter cluster is MCL2, which was exclusively composed of Cd-upregulated ABCGs/PDRs (Table 2). Among them, *ABCG36/OsPDR9* is reported induced rapidly and markedly in rice roots by Cd and Zn [46]. In rice, the Cd-inducible *OsABCG43/PDR5* is likely to sequester Cd at the subcellular level [47], and its Arabidopsis homologs, AtPDR12 and AtPDR8, are all involved in heavy metal resistance, and the Cd-inducible *AtPDR8* can confer Cd resistance as an extrusion pump [48]. Comparative transcriptomic analysis in two *Solanum* species with differential Cd accumulation suggests that the *Solanum PDR2*, which most closely related Arabidopsis gene is *AtPDR12*, may be involved in heavy metal resistance and transmembrane transport [6]. It is interesting to note that AtPDR12 and AtPDR8 also are members of the orthologs cluster MCL2.

Yet another interesting orthologs are ZIP transporters, which were synergistically downregulated by Cd stress, although they are dispersed in 4 orthologs clusters (Table 2). In plants, Cd is taken up and transported across plant membranes mainly by hijacking transporters for essential metals such as Zn, Fe, and Mn. For instance, members of the ZIP family are capable of transporting a variety of divalent cations such as Cd, Mn, Fe and Zn ions [49, 50]. Even in Zn/Cd-hyperaccumulators, Cd absorption from the soil is thought to occur mainly via ZIP family transporters [1]. For maize *ZIP* genes, they are suggested to be responsible for the uptake and translocation of Zn or Fe and involved in detoxification and storage of metals in plant cells [51].

Collectively, the similar expression pattern of these transporter orthologs in Cd-treated maize and rice roots could be as a consequence of the conserved function of orthologous genes. However, for the 3 types of transporters discussed above, only one ABCG (ZM2G391815) was mentioned as being Cd-responsive previously [19]. Therefore, further research is needed to determine the functional identity and precise roles of these promising Cd-modulated transporters in Cd tolerance.

### Conserved function of maize paralogs derived from gene tandem duplication

Gene duplication drives the evolution of novel functions, and plant genes involved in transcriptional regulation, signal transduction, and stress response tend to have paralogs [52–54]. Consistent with this hypothesis, the tandem gene duplication of heavy metal ATPase *HMA4*, which contributed to Zn/Cd hyperaccumulation in *Arabidopsis halleri*, was also occurred in another hyperaccumulator *Noccaea caerulescens* [55, 56]*.*

In the current study, it was interesting to observe that there were several blocks of paralogs co-modulated by Cd stress (Table 1). For instance, the members of two blocks of GLPs on chromosome 4 are independently sorted in tandem (Table 1). Importantly, tandem duplications appear to play an important role in expansion of the *GLP* family in rice and Arabidopsis [57]. It is known that GLPs can function as a cofactor for reinforcement of the cell wall through the production of H_2_O_2_ due to their SOD activity (e.g. OsGLP1, OsGLP2–1) [57–60]. Although no study has reported on the relationship between GLPs and the heavy metal tolerance in plant, the synchronized upregulation of GLP paralogs in Cd-treated maize roots is probably associated with their role in orchestrating Cd response.

Another tandem paralogs cluster is composed of two PALs on chromosome 5 (Table 1). Akin to this, 4 PAL genes were reported to be clustered on the same chromosome 2 [61]. PAL is the entry point enzyme directing the flow of reduced carbon to the various branches of phenylpropanoid metabolism, which products include soluble phenolics, flavonoids and the cell wall structural component lignin, all having diverse functions in plant development and response to abiotic and biotic challenges [61–63]. Furthermore, the phenylpropanoid pathway metabolites of yellow lupine roots could promote Pb stress tolerance [64].

Additionally, two patatin-like storage proteins are Cd-regulated tandem paralogs on chromosome 2, while the other two are reverse tandem paralogs on chromosome 1, and the latter two were also identified to be Cd-responsive in one previous report [19] (Table 1). It has been documented that patatin-like genes are involved in stress responses, hormone signaling, and development [65–67].

Yet another interesting tandem Cd-responsive paralogs are genes responsible for JA biosynthesis, since both 2 LOXs and 2 AOSs responsible for forming intermediate compounds in JA biosynthesis process are Cd-inducible and clustered on chromosome 1. The products of LOX enter oxylipin biosynthetic pathways where JAs are formed through the AOS branch [67–69]. JAs play multifunctional roles in regulation of tolerance against different environmental stresses including heavy metals [68, 70]. In heavy metal-treated plants, the concentration of JA was often elevated, thereby promoting the expression of genes involved in signaling pathways (such as ABA, ROS) and stress responses (e.g. transcription factors, antioxidant system, GSH biosynthesis) to alleviate heavy metal-induced toxicity in plants [70]. Among the 80 universal Cd-responsive DEGs, both ZmOPR2 and ZmOPR5 in cluster MCL166 (Table 1) were also significantly upregulated in both maize genotypes [19]. The synchronized expression patterns of *OPRs* and JA-responsive TFs genes have indicated that the JA signaling pathway is one of the crucial elements in the plant response to Cd stress [24, 71].

Taken together, several blocks of paralogs, such as GLPs, PALs and those involved in JA biosynthesis, displayed consistent co-expression pattern under Cd stress (Table 1). In this context, we proposed that these Cd-responsive paralogs contributed to Cd-tolerance, and their function might be conserved post gene tandem duplication.

### Common stress-responsive maize genes

In obvious manner, the regulation in hormone synthesis has been observed during heavy metal stress. It was well documented that the increased expression of *NCED*, the key enzyme for the synthesis of stress phytohormone ABA, was positively related to various stress tolerance [72], thus it was not surprising that the Cd-inducible *NCED* (ZM2G014392) is also one common stress-responsive maize gene (Table 3), which was identified in both two previous transcriptomic studies focusing on abiotic stresses responses [39, 40], but not mentioned as being Cd-responsive gene previously [19].

ABA and JA act synergistically in response to stress, and ethylene-responsive transcription factors (ERFs) are interesting targets for both JA-ABA interactions and ethylene [73]. In this investigation, the simultaneous upregulation of genes involved in biosynthesis of ABA (NCED), JA (LOX, AOS, AOC and OPR), and ethylene signaling (ERFs) suggests that these multiple-stress mediators coordinate the stress response in maize roots exposed to Cd (Table 1, Table 3), thus constitute a recurring theme of phytohormone crosstalk.

It was striking that the aforementioned metallochaperone ZmHIPP27 is one common stress-responsive gene (Table 3), and this is reminiscent of its rice ortholog OsHIPP42, which was one of the HIPPs associated with the response to a wide range of abiotic stresses including heavy metal (As, Cd, Cr, and Pb) toxicity [74]. HIPPs are unique to vascular plants and function in heavy metal homeostasis and regulating the transcriptional response to abiotic stresses and pathogens [41, 75]. Abiotic stresses inducible Arabidopsis AtHIPP26 interacts via its HMAD with the drought stress related zinc finger transcription factor ATHB29 [76]. There is substantial evidence that HIPPs can have a role in Cd-detoxification, possibly by binding Cd [43]. Moreover, the plants overexpressing *AtHIPP26* were more tolerant to Cd treatment than wild type [45]. Therefore, this common stress-responsive ZmHIPP27, the ortholog of abiotic stresses inducible *AtHIPP26* and *OsHIPP42,* together with the latter rice ortholog, provide a new avenue to further investigate the molecular mechanism beneath the Cd stress response in crop plants.

Of particular interest is the rice SNAC1, which overexpression improves both drought and salt tolerance in rice and cotton [77, 78], thus it has been alluded to be common stress-responsive [22, 77]. In the present study, SNAC1 and its ortholog in maize were identified to be common stress-responsive as well as Cd-inducible genes (Table 3). ZmSNAC1 has been described as a stress-responsive factor acting in positive modulation of abiotic stress tolerance, and ZmSNAC1 confers enhanced tolerance to dehydration in transgenic Arabidopsis [79].

These results collectively suggest that these orthologous genes, simultaneously modulated in maize and rice roots exposed to Cd treatment are also common stress-responsive genes in both species. However, the majority of the 30 common stress-responsive genes except ClpD and ZmPK1 were not mentioned in previous Cd-stressed maize transcriptomic study (Table 3) [19]. Thus, the present transcriptomic analysis provides novel valuable information about the conserved Cd response from the viewpoint of common stress-responsive genes, such as ZmHIPP27 and SNAC1, which may facilitate further investigations on the Cd-tolerance mechanisms.

### The key enzyme for GABA biosynthesis GAD contributed to cd tolerance

GABA-regulated processes are thought to include developmental regulation, stress tolerance, carbon:nitrogen balance, and long-distance transport [80]. The rapid accumulation of GABA during biotic and abiotic stresses is well documented [81–84]. For instance, heavy metals (e.g. Cu, Zn and Cr) stimulated the accumulation of GABA [85, 86]. GABA works in harmony with phytohormones and the regulation of phytohormones by exogenous GABA could play a key role in combating plant stress [87]. These collectively indicate GABA as one common stress signal [82]. Therefore, exogenous GABA enhances plant resistance to some stresses. For instance, GABA treatment might protect rice plants against the deleterious effects of ammonium toxicity [88]. GABA also imparts partial protection from salt stress injury to maize seedlings [89].

Further investigations suggested that exogenous application of GABA resulted in a significant increase in endogenous GABA concentration, and this accumulation of GABA was associated with the activity of GAD, the key enzyme catalyzing the decarboxylation of glutamate to GABA [82, 84, 90–92].

Overexpressing *SlGAD3* in tomato fruits led to an increase in GABA levels at the red-ripe stage [92]. Rice plants overexpressing *OsGAD* gene were able to accumulate GABA under short-term salinity [93]. Several GAD genes were upregulated at the mRNA level and this is subsequently reflected in an increase in GABA at the metabolite level in tobacco under drought stress [94].

In the current study, overexpression of maize ZmGAD1 and ZmGAD2 in Cd-sensitive yeast and tobacco leaves *in planta* all enhanced Cd tolerance of the host cells (Figs. 2, 3). These findings implicated that GADs participate in the accumulation of GABA, which contribute to stress acclimation or alleviation.

## Conclusions

In summary, the ortholog analysis of Cd-treated maize and rice transcriptomes uncovered 880 orthologs groups, and the highlight was the discovery of Cd co-modulated orthologs in this two cereal crops. Further comparative investigation revealed that ~ 30 maize Cd-responsive genes with rice counterparts were also common stress-responsive genes such as heavy metal-associated domain (HMAD) isoprenylated protein ZmHIPP27, stress-responsive transcription factor ZmSNAC1, and vp14 (one NCED for ABA biosynthesis). These collectively suggest that the network underlying Cd stress responses and tolerance, which different plant species have developed to adapt to other stresses, could assist plants to acclimate to diverse stresses. Importantly, the orthologs of GAD, the key enzyme for GABA synthesis, were concomitantly upregulated in maize and rice roots exposed to Cd treatment. Moreover, maize GADs confer Cd tolerance in yeast and tobacco leaves *in planta* via heterologous expression. Notably, the aforementioned several promising Cd-upregulated genes (e.g. *ZmHIPP27*, *ZmSNAC1*, *vp14* and *ZmGADs*) with rice counterparts were identified to be novel Cd-responsive genes in maize. This study extends the understanding of the common molecular mechanisms of plant roots response to Cd and other abiotic stresses, and will be useful for deciphering major candidate genes for improving Cd tolerance in cereal plants.

## Additional files

Additional file 1: **Table S1.** RNAseq information of maize roots under Cd stress. (XLSX 9 kb)

Additional file 2: **Table S2.** Primers used for quantitative RT-PCR and cloning maize GADs. (XLSX 10 kb)

Additional file 3: **Table S3.** Cd-responsive 5166 DEGs in maize roots post Cd treatment. (XLSX 513 kb)

Additional file 4: **Table S4.** Quantitative RT-PCR of 10 random selected DEGs expression in maize roots exposed to Cd. (XLSX 10 kb)

Additional file 5: **Table S5.** Cd-responsive 2567 DEGs in rice roots post Cd treatment. (XLSX 268 kb)

Additional file 6: **Table S6.** Eight hundred eighty plant orthologous groups composed of Cd-responsive orthologs in maize and rice. (XLSX 78 kb)

Additional file 7: **Figure S1.** Global view of Cd-responsive maize orthologs with rice counterparts in metabolic pathways. For the 1074 maize DEGs with rice orthologs, 471 data points were visible on the metabolic pathways (Arabidopsis seed-Molecular Networks) using MapMan software. The colored boxes indicate the Log2 ratio of Cd1h/ck1h. (PDF 1258 kb)

## Abbreviations

ABC: ATP-binding cassette
AOS: Allene oxidase synthase
ClpD: Chloroplastic caseinolytic protease
DEG: Differentially expressed gene
ERF: Ethylene-Response Factor
GABA: γ-aminobutyric acid
GAD: Glutamate decarboxylase
GLP: Germin-like protein
HIPP: HMAD isoprenylated plant protein
HMA: Hheavy metal ATPase
HMAD: Heavy metal associated domain containing protein
HSF: Heat shock transcription factor
JA: Jasmonic acid
LOX: Lipoxygenase
MATE: Multidrug and toxic compound extrusion
NCED: 9-cis-epoxycarotenoid dioxygenase
OPR: 12-oxo-phytodienoic acid reductase
PAL: Phenylalanine ammonia-lyase
PDR: Pleiotropic drug resistance
SNAC1: Stress-responsive NAC 1
ZFP: Zinc finger protein
ZIP: ZRT- and IRT-like protein
