# Supplementary figures and images for: Comparative analysis of Cd-responsive maize and rice transcriptomes highlights Cd co-modulated orthologs
Source: BMC Genomics. 2018 Sep 26;19:709. doi: 10.1186/s12864-018-5109-8 (PMC6158873; doi:10.1186/s12864-018-5109-8)

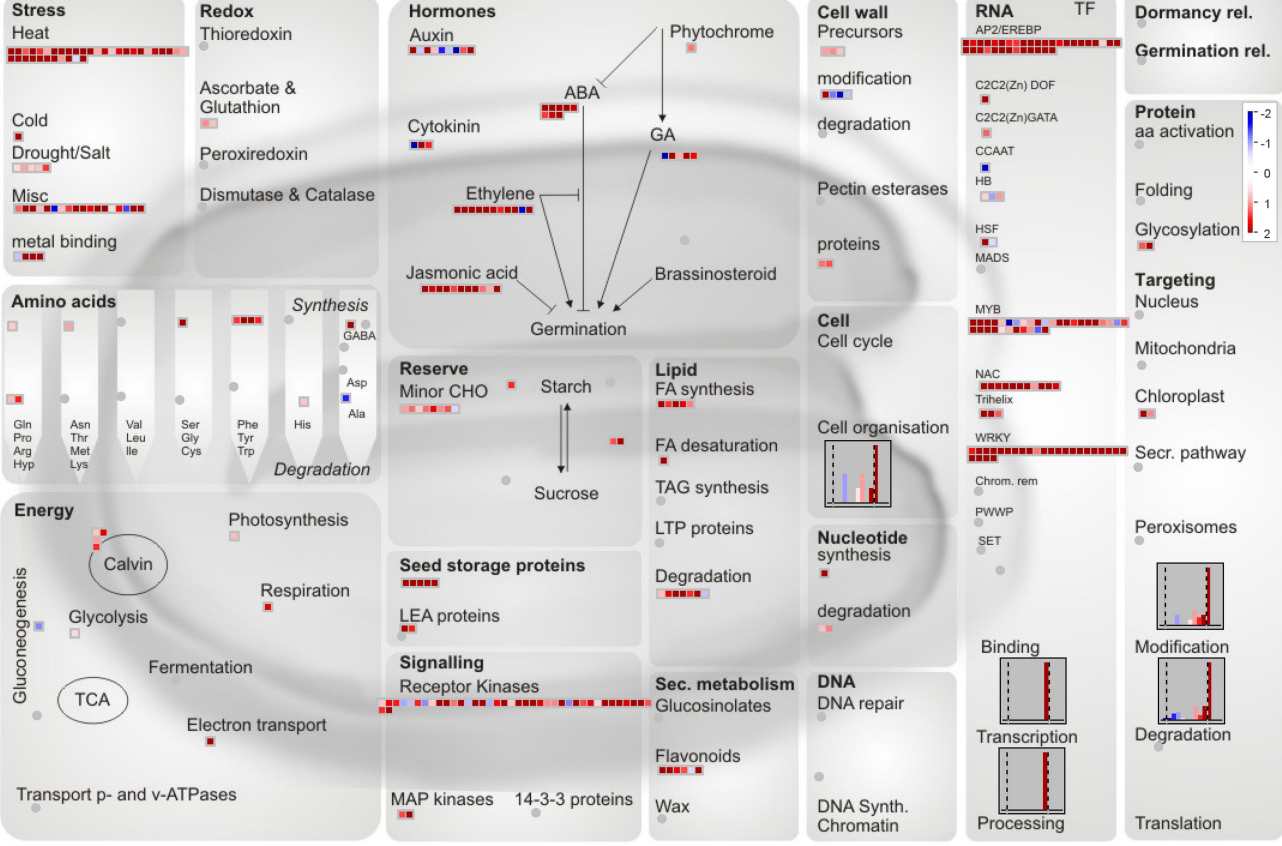

Supplement: Supplementary file 7 — Figure S1. Global view of Cd-responsive maize orthologs with rice counterparts in metabolic pathways. For the 1074 maize DEGs with rice orthologs, 471 data points were visible on the metabolic pathways (Arabidopsis seed-Molecular Networks) using MapMan software. The colored boxes indicate the Log2 ratio of Cd1h/ck1h. (PDF 1258 kb) [file 12864_2018_5109_MOESM7_ESM.pdf]
